# Serum Removal from Culture Induces Growth Arrest, Ploidy Alteration, Decrease in Infectivity and Differential Expression of Crucial Genes in *Leishmania infantum* Promastigotes

**DOI:** 10.1371/journal.pone.0150172

**Published:** 2016-03-09

**Authors:** Pedro J. Alcolea, Ana Alonso, Miguel A. Moreno-Izquierdo, María A. Degayón, Inmaculada Moreno, Vicente Larraga

**Affiliations:** 1 Department of Molecular Microbiology and Biology of Infections, Centro de Investigaciones Biológicas, Consejo Superior de Investigaciones Científicas (CIB-CSIC), Madrid. Spain; 2 Unit of Microbial Immunology, Centro Nacional de Microbiología, Virología e Inmunología Sanitarias, Instituto de Salud Carlos III (CNM-ISCIII), Majadahonda, Spain; National Center for Cell Science, INDIA

## Abstract

*Leishmania infantum* is one of the species responsible for visceral leishmaniasis. This species is distributed basically in the Mediterranean basin. A recent outbreak in humans has been reported in Spain. Axenic cultures are performed for most procedures with *Leishmania* spp. promastigotes. This model is stable and reproducible and mimics the conditions of the gut of the sand fly host, which is the natural environment of promastigote development. Culture media are undefined because they contain mammalian serum, which is a rich source of complex lipids and proteins. Serum deprivation slows down the growth kinetics and therefore, yield in biomass. In fact, we have confirmed that the growth rate decreases, as well as infectivity. Ploidy is also affected. Regarding the transcriptome, a high-throughput approach has revealed a low differential expression rate but important differentially regulated genes. The most remarkable profiles are: up-regulation of the GINS Psf3, the fatty acyl-CoA synthase (FAS1), the glyoxylase I (GLO1), the hydrophilic surface protein B (HASPB), the methylmalonyl-CoA epimerase (MMCE) and an amastin gene; and down-regulation of the gPEPCK and the arginase. Implications for metabolic adaptations, differentiation and infectivity are discussed herein.

## Introduction

*Leishmania infantum* is the etiological agent of zoonotic visceral leishmaniasis in the Mediterranean basin, where dogs are the main reservoirs. A recent outbreak in humans has been described in Spain [1–3], where the main vector is *Phletobomus perniciosus* (Psychodidae: Phlebotominae) [4]. Sand flies are the blood-feeding vector hosts in the life cycle of the parasite. Promastigote development takes place within the gut of the sand fly simultaneously to migration towards the anterior gut, whereas blood components are progressively digested, leading to nutrient depletion [5–7]. Chemotaxis and osmotaxis promote directed migration [8]. After development, promastigotes are released into the dermis of the mammalian host during blood feedings. Then, they differentiate to the amastigote stage within host phagocytic cells. Eventually, a sand fly feeds from the infected mammalian host and amastigotes are released to the mid gut, where they become motile promastigotes.

The promastigote stage is generally cultured in complex undefined liquid media at 26–27°C, which mimics to some extent the conditions of the sand fly gut microenvironment [9–14]. Mammalian serum provides complex nutrients in cultures, thus improving growth kinetics. Inactivation of serum is performed by heating at 56°C for 1 h. This procedure avoids lysis of promastigotes by proteins of the complement system. The parasite is metabolically versatile because it is able to use amino acids, fatty acids or glucose as the major carbon source [15] and consequently, adapt to different environments. However, culture may affect differentiation in some aspects [16]. The aim of this study is the evaluation of general and specific consequences of serum deprivation for cultured promastigotes, including growth rate, ploidy, infectivity and differential gene expression.

## Materials and Methods

### Promastigote cultures

The *L*. *infantum* MCAN/ES/98/10445 (zymodeme MON-1) isolate was used in this study. Promastigotes were cultured in triplicate at 27°C in complete medium (CM) or in heat inactivated fetal bovine serum (HIFBS)-depleted medium for 4 days. CM consists of RPMI 1640 supplemented with 2 mM glutamine (Lonza, Karlskoga, Sweden), 10% HIFBS (Lonza) and 100 UI/ml penicillin-100 μg/ml streptomycin (Life Technologies, Carlsbad, CA). Cell recovery from cultures was performed by centrifugation at 2,000g for 10 min. Morphology was routinely evaluated at the light microscope (40X). For this purpose, 10^7^ promastigotes were harvested, washed in PBS and resuspended in 1 ml PBS. A 10 μl aliquot was deposited between a slide and a coverslip. Cell counting was performed at the light microscope in a Neubauer chamber (40 X) after diluting 20 times an aliquot of promastigote culture in 0.5 M EDTA. Differences in growth of HIFBS-depleted and CM promastigote cultures were compared by the Student's t-test.

### Cell cycle analysis by flow cytometry

Samples of 50 x 10^6^ promastigotes were harvested for cell cycle analysis. Three biological replicates of the experiment were performed. G1 arrest was achieved by 6h treatment with 0.8 mg/ml hydroxyurea in fresh CM or HIFBS-depleted medium (Sigma-Aldrich, Basel, Switzerland). Thereafter, the cells were centrifuged, washed three times with PBS and fixed with 1 ml cold 70% ethanol at -20°C for 30 min. Next, promastigotes were harvested, washed twice with PBS and incubated for 30 min in 0.5 ml of a solution containing 50 μg/ml propidium iodide (PI) and 100 μg/ml RNase A (Sigma-Aldrich) in PBS. PI uptake was analyzed in a FACSCalibur^™^ flow cytometer using CELLQuest^™^ software (Becton Dickinson, Franklin Lakes, NJ) by gating promastigotes at forward-angle versus side-angle light scatter and registering fluorescent emission collected in the FL2-A detector through a 585 nm band pass filter.

### Evaluation of *in vitro* infection of stimulated U937 cells with promastigotes.

The non-adherent human myeloid cell line U937 (ATCC^®^ CRL1593.2), originally obtained from pleural effusions of a patient with histiocytic leukemia [17], was cultured at 37°C in complete medium in the presence of 5% CO_2_. After 72 h, 2 x 10^5^ cells/cm^2^ were harvested at 250g for 10 min and stimulated with 20 ng/ml phorbol 12-myristate 13-acetate (Sigma-Aldrich) [18] in CM over 8-well cell chamber slides (LabTek, New York, NY). This treatment allows differentiation to macrophage-like cells. Then, the wells were mildly rinsed with CM and the infection step performed by incubating cells at 37°C for 2 h in 400 μl of CM containing *L*. *infantum* promastigotes. The promastigote:cell ratio was 5:1. Next, cells were washed three times with CM to remove remaining promastigotes. Infected cells were then incubated in CM at 37°C, 5% CO_2_ for 72 h. Three final washes were performed before treatment with hypotonic solution (180 μl CM diluted with 220 μl water per well) for 5 min. Four washes were carried out with 150 μl ethanol-acetic acid 3:1 after removing the hypotonic solution. Fixation was carried out with the same solution for 10 min and this step was repeated three times. Finally, cells were allowed to air dry and the wells removed from the slide. Staining was performed with Diff-Quick^®^ Stain Solution I and II (Dade Behring, Marburg, Germany). The preparations were washed with distilled water, air dried and mounted with Entellan^®^ Neu (Merck, Darmstadt, Germany). The average infection rate and the number of amastigotes per infected cell were assessed at the light microscope (40X) and contrasted by the Student’s t-test.

### Isolation of total RNA and protein

Total RNA extractions were immediately performed with TRizol^®^ reagent (Life Technologies) following the manufacturer´s instructions.

Whole protein was obtained from 10^8^ cells by mild agitation at 4°C during 30 min in 50 mM Tris-HCl pH 7.4, 2 mM EDTA and 0.2% TritonX-100 in the presence of a cocktail of protease inhibitors (Roche, Mannheim, Germany). Then, samples were centrifuged at 8,000g at 4°C for 10 min. Protein concentration was estimated by the Bradford method.

### Western blot

SDS-PAGE of protein extracts was performed at 12 mA for 30 min, then at 30 mA for 90 min, in 8% slab gels casted in a MiniProtean II Cell system (BioRad, Hercules, CA). Twenty μg protein extract was loaded per well including 1 μl Benzonase Nuclease HC (Novagen, Madison, WI). Proteins were then transferred to 0.45 μm nitrocellulose membranes (BioRad) at 100 V for 1 h in a Mini Trans-Blot Cell wet transfer system (BioRad). Then, the membranes were blocked with 5% skimmed milk in PBS-0.1% Tween 20 (Sigma) for 1 h and washed three times with PBS-0.1% Tween 20 for 15, 5 and 5 min respectively. After that, membranes were incubated with 1∶500 of rabbit anti-LACK polyclonal serum diluted 1:500 in blocking solution for 2 h [19]. A monoclonal mouse anti-*L*. *mexicana* gGAPDH antibody kindly provided by Paul Michels (University of Edinburg) was also included in the mixture at 1:10,000 dilution [20]. The washing steps were repeated. Next, 90 min incubation was carried out with 1∶2,000 HRP-conjugated goat anti-rabbit IgG (DAKO, Ely, UK) and the membrane was washed again. Finally, ECL-based detection (GE Healthcare, Pittsburg, PA) and development was performed in X-ray film according to the manufacturer's instructions. Densitometry was performed with Gel Doc XR System and Quantity One version 4.6. software (BioRad) and the intensity data of both groups were compared by the Student's t-test including three biological replicates.

### Microarray hybridization analysis

Differential gene expression was analyzed by shotgun DNA microarray hybridization experiments as described [21]. Briefly, mRNA was amplified from three replicate samples using MessageAmp^™^ II aRNA Amplification Kit (Life Technologies) and cyanine-labelled cDNA was synthesized (Life Technologies). Then, combined Cy5/Cy3 cDNA microarray hybridizations were carried out in triplicate (HIFBS-depletion/CM). The slides were scanned with a GenePix 4100A instrument (Axon, Foster City, CA) and raw medians of fluorescence intensity values obtained were normalized by the LOWESS per pin method. Differential transcript abundance was inferred by the paired Student’s t test. The spot selection criteria were: fold-change (F) > 1.7 (Cy5/Cy3; Cy5 > Cy3) or < -1.7 (-Cy3/Cy5; Cy5 < Cy3); fluorescence intensity value 10-fold higher than the substracted background; p < 0.05 [21]. Clones that fulfilled these cutoff values were recovered from the genomic library that was used for microarray construction. The clone ends sequenced with the M13 universal primers and they were assembled by alignment with the whole-genome *L*. *infantum* sequence as detailed in [21]. Depending on mapping and assembling outcomes, clones were classified in type a (congruent alignments, unique pair of alignments), b (congruent alignments, more than one pair of alignments) and c (uncongruent alignments or lack of one insert end read) [21].

### Real time quantitative RT-PCR analyses (qRT-PCR)

Unlabelled single-stranded cDNA was synthesized following the same procedure as for labelled cDNA but using a mixture of unlabelled dNTPs (10 mM each). Custom TaqMan^®^ FAM-MGB Assay-by-Design primers and probes (Life Technologies) are provided in the S1 Table and amplification with TaqMan^®^ Universal Master Mix 2x (Life Technologies) was run in a 7900HT Fast Real Time PCR system using SDS 3.1 software (Life Technologies) following the manufacturer’s instructions. The gGAPDH was the reference gene and the fold-change values were calculated with efficiency-corrected normalized quantities [22].

### BCAT activity assay

The assay was performed with the substrate 3-methyl-2-oxopentanoate and L-glutamate was added as the co-substrate. All reagents and enzymes were purchased from Sigma-Aldrich. Incubations were carried out in quadruplicate at 25°C for 30 min in 1ml buffered (0.1M Tris-HCl, pH8.3) reaction mixture containing 2.1 mM 3-methyl-2-oxopentanoate, 300 mM L-glutamate, 0.2 mM NADH, 0.1 mM pyridoxalphosphate (PLP), 200 mM L-aspartate, 200 μmol/min/l L-aspartate aminotransferase (ASAT), 1 mmol/min/l L-malate dehydrogenase (MDH) (both enzymes from porcine heart) [23] and 150 μg protein extract. Negative controls without NADH were also set. The time course consumption of NADH was monitored by measuring the decay of absorbance at 334 nm continuously with a Cary 4000 spectrophotometer (Agilent Technologies, Santa Clara, CA). The results were contrasted by the paired Student's t test.

## Results

### Serum depletion considerably affects growth rate and ploidy

**Fig 1:**
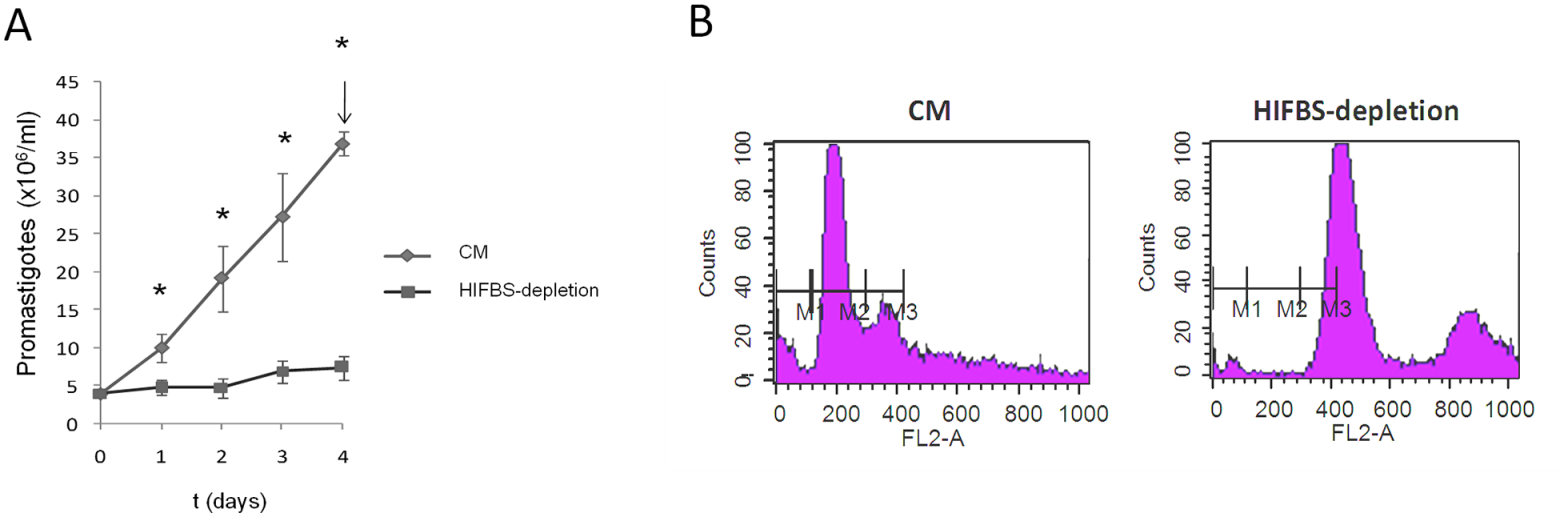
Growth monitoring and cell cycle analysis of *L*. *infantum* HIFBS-depleted promastigotes and CM promastigotes. (A) Average growth curves of three replicate cultures of HIFBS-depleted and CM *L*. *infantum* promastigotes. Cell density was registered daily in a Neubauer chamber at the light microscope (40 X). The differences between the HIFBS-depletion and CM groups are statistically significant (Student's t-test, p* < 0.01) at all time points. (B) Cell cycle analysis of HU-treated *L*. *infantum* HIFBS-depleted and CM promastigotes by PI-based flow cytometry. One out of three biological replicates is represented for both conditions. Propidium iodide fluorescence intensity was registered by flow cytometry through the FL2-A detector (585 nm). As expected, the G1 (markers M1-M2) and G2 (M2-M3) peaks of CM promastigotes are centered at 200 and 400 fluorescence intensity units, respectively. In the case of HIFBS-depleted promastigotes, these peaks are displaced to higher values in the FL2-A axis as they do not fit between M1, M2 and M3. In fact, the values are approximately twice the CM values, which indicates that the population is tetraploid.

As expected, pronounced decrease of proliferation is observed when the promastigote culture does not contain HIFBS (Fig 1A). Changes in promastigote morphology were not detected, except for more frequent lack of elongation of the fusiform cell body in most HIFBS-depleted promastigotes. Tetraploidy was observed also in this case (Fig 1B, S2 Table).

### *In vitro* infection ability of HIFBS-depleted and CM *L*. *infantum* promastigotes

**Fig 2:**
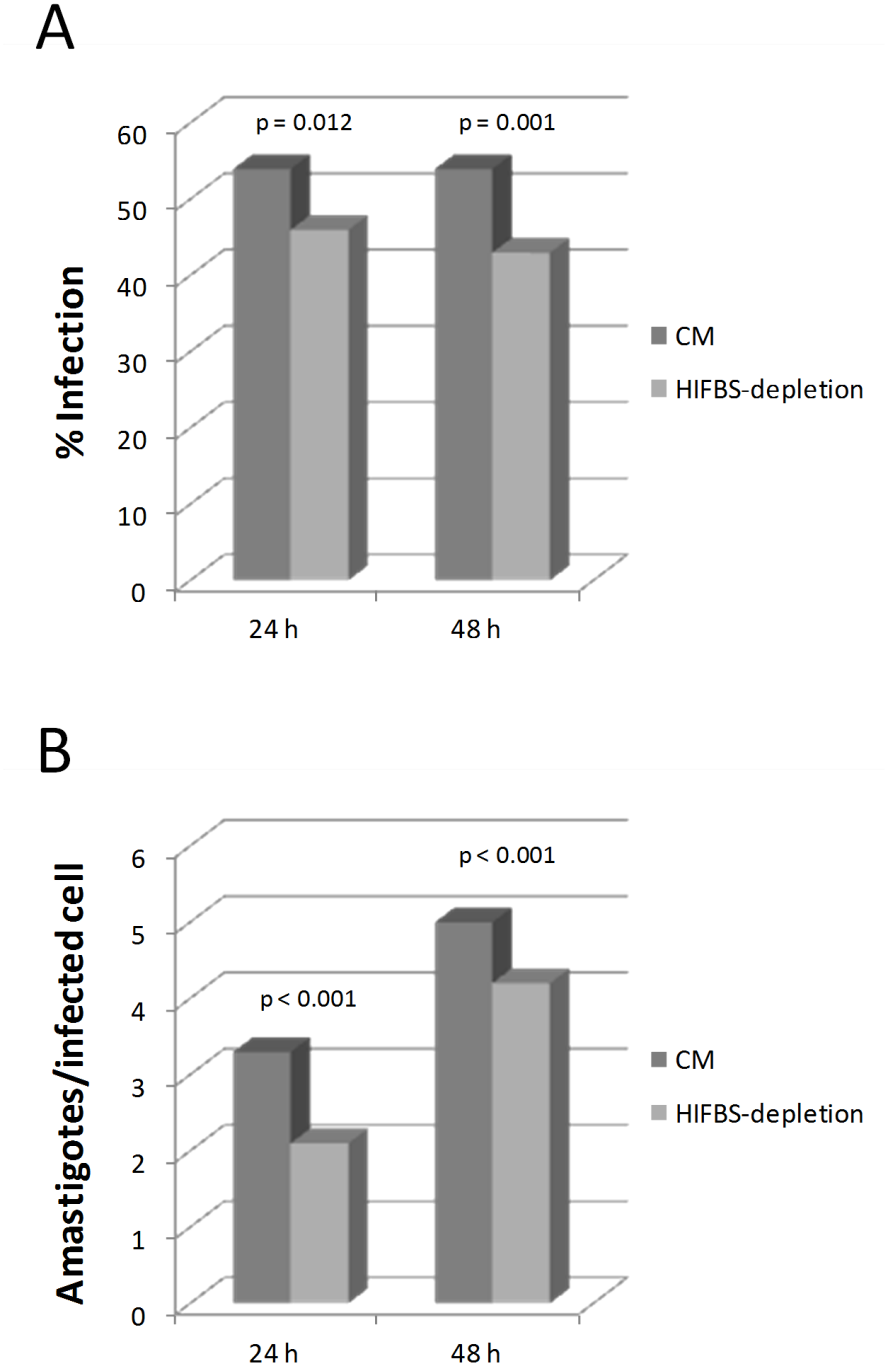
Evaluation of *in vitro* infectivity of *L*. *infantum* promastigotes in stimulated U937 cell cultures. The U937 cell line was differentiated with phorbol esters and *in vitro* infected with *L*. *infantum* CM and HIFBS-depleted promastigotes at a phagocyte:promastigote ratio 1:20. The null hypothesis contrasted with the paired t-test was equal infectivity of HIFBS-depleted and CM promastigotes measured in triplicate as percentage of infected cells or number of amastigotes per infected cell. It was contrasted by the paired Student’s t-test (p-values provided in the graph). (A) Infection rate measured in terms of percentage of infected cells 24 and 48 h post-infection. Mean ± SD: 54 ± 1 (CM, 24 h); 54 ± 4 (CM, 48 h); 46 ± 3 (HIFBS-depletion, 48 h); 43 ± 1 (HIFBS-depletion, 48 h). (B) Average number of amastigotes per infected cell measured 24 and 48 h post-infection. Mean ± SD: 3.3 ± 0.3 (CM, 24 h); 5.0 ± 0.0 (CM, 48 h); 2.1 ± 0.1 (HIFBS-depletion, 48 h); 4.2 ± 0.1 (HIFBS-depletion, 48 h).

The average infection rate and the number of amastigotes per infected U937 cell were evaluated at 24 and 48 h after infection with CM and HIFBS-depleted promastigotes. The average infection rate is 54% with CM and 43% with HIFBS-depleted promastigotes after 24 and 48 h (Fig 2A). Cells infected with CM promastigotes contained 3.3 ± 0.3 amastigotes at 24 h and 5.0 ± 0.0 at 48 h, whereas the results were 2.1 ± 0.1 at 24 h and 4.2 ± 0.1 at 48 h in the case of cells infected with HIFBS-depleted promastigotes (Fig 2B). These differences are statistically significant according to the paired Student's t-test outcome. Therefore, serum depletion decreases the infection ability of promastigotes in terms of infection rate and number of amastigotes per infected cell.

### Serum depletion slightly affects gene expression at the transcript level in *L*. *infantum* promastigotes

**Fig 3:**
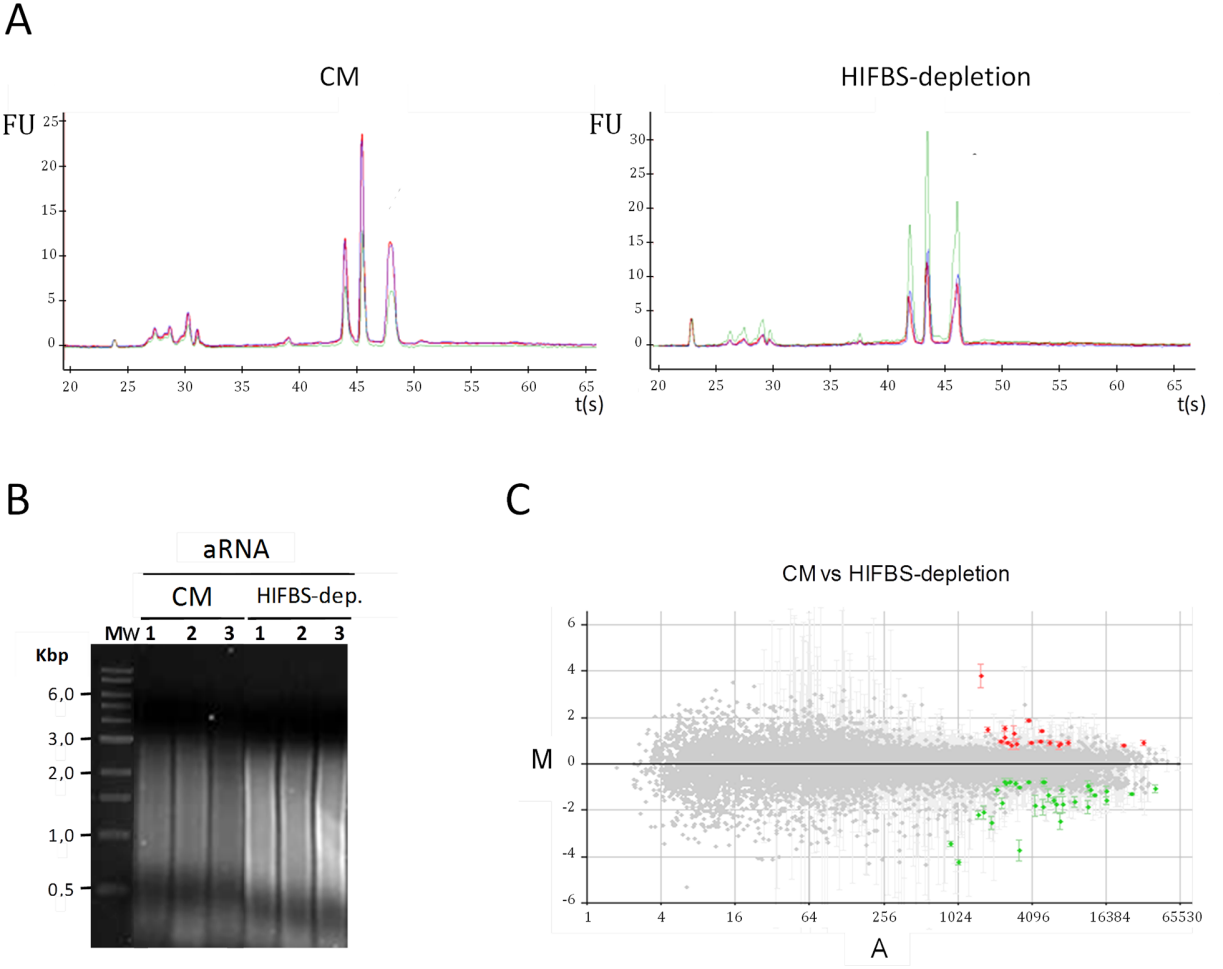
Genome microarray hybridization analysis for comparative expression profiling of HIFBS-depleted versus CM *L*. *infantum* promastigotes. (A) Electropherograms (FU, fluorescence units vs. time in seconds) of HIFBS-depleted and CM promastigote total RNA triplicate samples obtained by capillary electrophoresis with the Agilent 2100 Bioanalyzer. (B) 1% agarose gel electrophoresis of amplified mRNA (aRNA) from total RNA samples of *L*. *infantum* HIFBS-depleted and CM promastigotes. (C) Average M/A scatter plots of the HIFBS-depletion/CM three-replicate microarray hybridization analysis. M = (log_2_Ri_log_2_Gi) and A = [(log_2_Ri + log_2_Gi)/2], where R and G are, respectively, red (Cy5-cDNA from HIFBS-depleted promastigotes) and green (Cy3-cDNA from CM) intensity values. Red spots correspond to selected DNA fragments containing a gene up-regulated at least 1.7 times and green spots represent those down-regulated at least 1.7 times. Further criteria for spot selection are detailed in the text. The SD values are displayed in the scatter plot. Differential expression was contrasted by the Student's t-test for each individual clone with the AlmaZen software. The statistically significant differences are highlighted in red and green as mentioned above.

**Fig 4:**
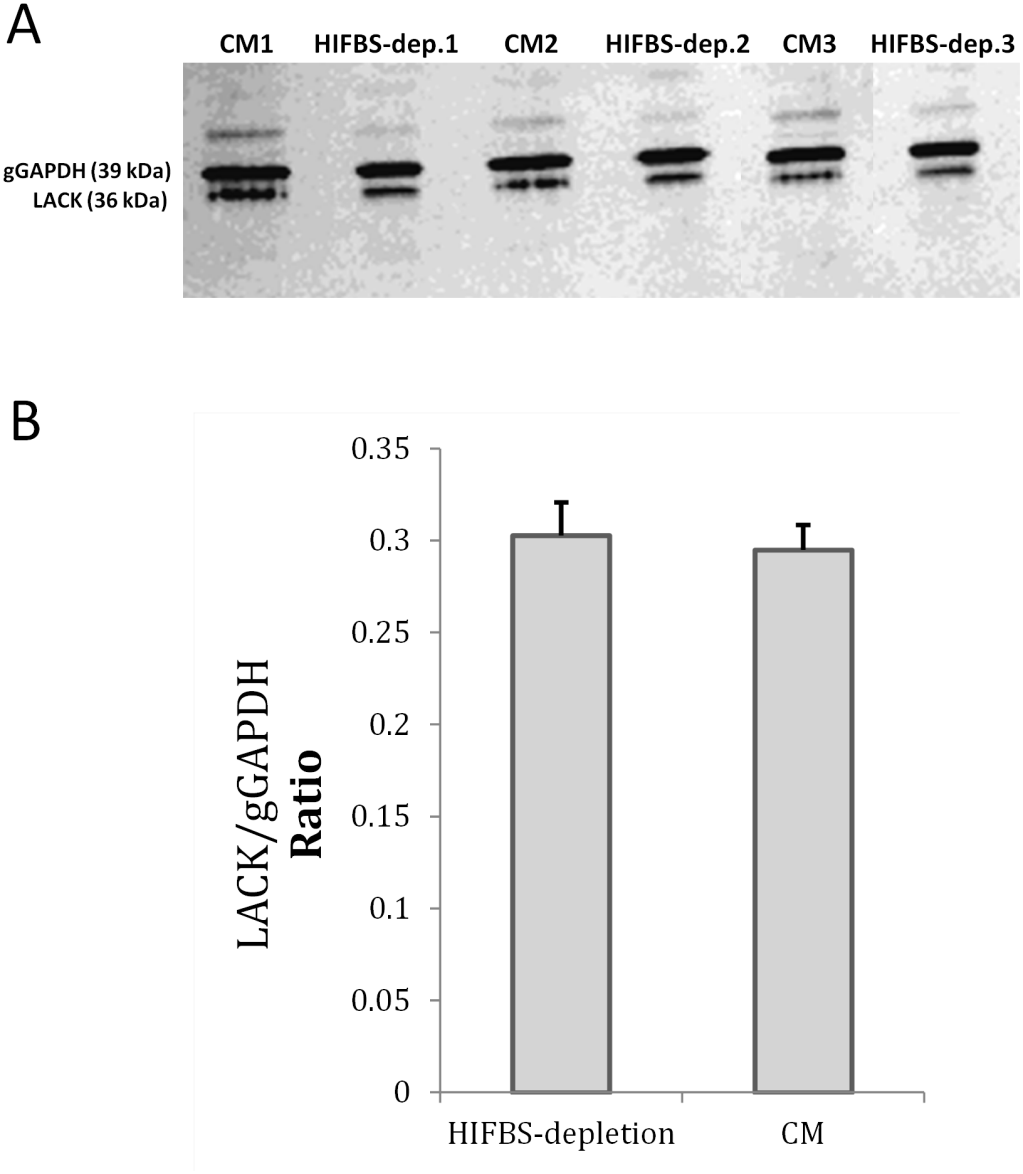
LACK expression is maintained under serum depletion. (A) Evaluation of relative abundance of the LACK antigen by Western blot performed with the anti-LACK polyclonal antibody (diluted 1:500) over total protein extracts of *L*. *infantum* promastigotes grown under HIFBS-depletion or in CM. The anti-gGAPDH polyclonal antibody (diluted 1:10,000) was used as the loading control antibody. The secondary antibody was HRP-conjugated goat anti-rabbit IgG (diluted 1:2,000). Chemoluminescence detection was performed with the ECL reagents (GE Healthcare) and the membrane was developed in an X-ray film. Upper bands are likely aggregates of the LACK protein [19]. (B) Bar graph of the relative expression ratio of the LACK antigen with respect to the gGAPDH. Mean and SD of three biological replicates are represented. Densitometry was performed with Gel Doc XR System and Quantity One version 4.6. software (BioRad). Both groups were compared by the Student's t-test including three biological replicates.

Total RNA isolated from HIFBS-depleted and CM promastigotes was not degraded (Fig 3A). mRNA from all samples was successfully amplified (Fig 3B). Differential expression of the control genes included in the microarrays [21] was not detected (S3 Table). The A2 gene is not differentially regulated and the flagellum remains emergent from the cell body. These facts indicate that HIFBS-depleted promastigotes do not undergo the developmental process to the amastigote stage, as well as CM control promastigotes. Also, expression of the LACK antigen remains constant in promastigotes at the transcript (S3 Table) and protein (Fig 4) levels when they are depleted from serum.

**Fig 5:**
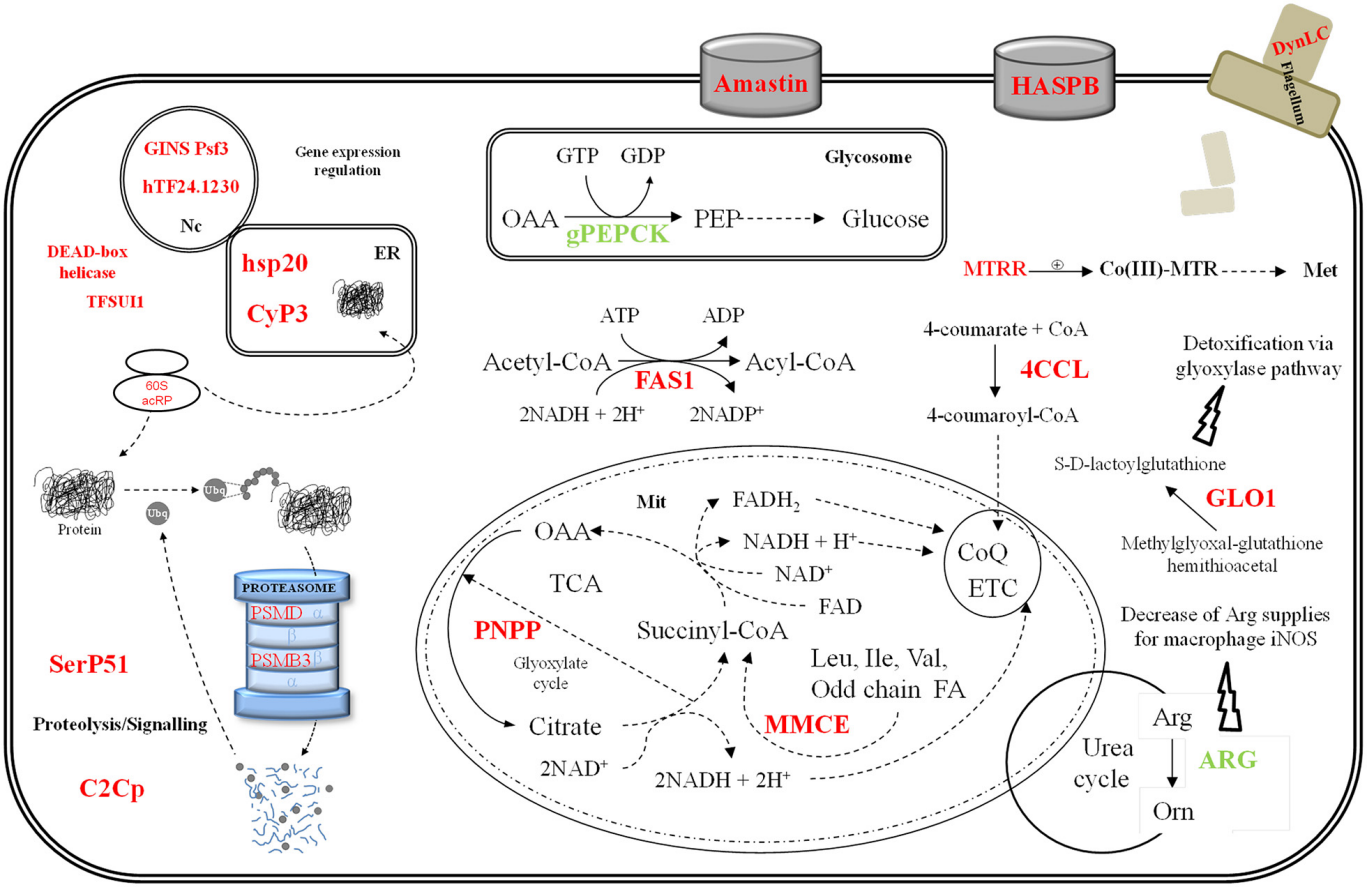
Differential gene expression triggered by serum depletion in culture. Color legend: red, up-regulation in HIFBS-depleted promastigotes/CM; green, down-regulation. Genes involved in DNA repair, gene expression, protein folding, proteolysis, detoxification, signalling, the flagellum and the surface coat are up-regulated under serum depletion. The gene LinJ.31.1680 encodes the GINS complex protein subunit Psf3, which is involved in the initiation of DNA replication. GINS is a component of the eukaryotic replicative helicase essential for the establishment of DNA replication forks (reviewed in [51]). The transcript levels of this gene are more reduced in HIFBS-depleted promastigotes than in CM promastigotes. The hypothetical protein LinJ.24.1230 (hTFLi24.1230; sequence-specific DNA binding transcription factor activity GO0003700), the translation factor TFSUI1 and the DEAD-box helicase-like protein are up-regulated in HIFBS-depleted promastigotes, whereas the helicase is down-regulated. Two proteins involved in protein folding are also up-regulated (a cyclophilin and the heat shock protein 20, hsp20). Four genes involved in proteolysis are over-expressed: a calpain-like cysteine peptidase (C2Cp), a serine peptidase E, family 51 (SerP51), the proteasome non-ATPase regulatory subunit 8 (PSMD8) and the proteasome β subunit 3 (PSMB3). PSMB3 and PSMD8 are involved in protein degradation via the ubiquitin-proteasome system. The surface molecule-encoding genes hydrophilic surface protein B (HASPB) and amastin-like LinJ.34.2660 are up-regulated, as well as the glyoxylase I (GLO1), one of the components of the glyoxylase detoxification system.

The differential gene expression rate is 0.4% (33 genes out of 8154 coding genes [24]) in HIFBS-depleted promastigotes with respect to CM (Fig 3C, Table 1). Genes involved in DNA repair, gene expression regulation, protein folding, proteolysis, metabolism, detoxification, signalling, the flagellum and the surface coat are up-regulated in HIFBS-depleted promastigotes (Fig 5, Table 1). Differential regulation of five genes was validated by qRT-PCR, which also solved two clones that represent more than one gene sequence (Table 1). By contrast, two genes tested by qRT-PCR are negative for differential expression (clones Lin138C1 and Lin309D1). Consequently, at least one of the remaining genes represented in these clones are differentially regulated.

**Table 1:** Differentially regulated genes of known function in HIFBS-depleted promastigotes. The features described are: number of clone; fold change (F ≥ 1.7, up-regulation; F ≤ -1.7, down-regulation); log_2_F and standard deviation (SD); Student's t-test, *p*; multiple sequence alignment expect value (e-value); annotation; annotated gene sequence; and qRT-PCR outcome (Student's t-test, p* < 0.05). N.D.: not determined. Genes in italics (clones that overlap with more than one annotated gene): they are not differentially regulated (confirmed by qRT-PCR) or there is no evidence to support that they are differentially regulated in other cases (not determined by qRT-PCR).

*Clone*	*F*	*Log*_*2*_*F± S*	*p*	*e-value*		*Def*.	*Annotation*	*Anotated gene function*	*qRT-PCR*	
				*Fw*	*Rv*					*F ± SD*
Lin7C3	1.80	0.9 ± 0.1	0.023	0	0	c	*LinJ*.*06*.*0460*	*Hypothetical protein*, *conserved*	*N*.*D*.	
Lin8D4	1.98	1.0 ± 0.1	0.009	0	0	c	*N*.*A*.	*N*.*A*.	*N*.*A*.	
Lin12H3	-2.5	-1.4 ± 0.2	0.006	0	0	b	LinJ.35.1490	Arginase, putative	+	-3.3 ± 0.4*
Lin17G12	2.73	1.4 ± 0.1	0.025	0	0	a	LinJ.26.0030	Methylmalonyl-CoA epimerase	+	10.0 ± 0.5*
Lin19B1	1.87	0.9 ± 0.1	0.021	0	0	b	LinJ.22.0580	Hypothetical protein, conserved	N.D.	
Lin24C4	1.86	0.9 ± 0.3	0.026	0	0	a	LinJ.36.4950	Methionine synthase reductase	N.D.	
Lin32A12	1.70	0.8 ± 0.2	0.013	0	0	b	*LinJ*.*30*.*1640*	*Hypothetical protein*, *conserved*	*N*.*D*.	
							*LinJ*.*30*.*1650*	*Hypothetical protein*, *conserved*	*N*.*D*.	
							*LinJ*.*30*.*1660*	*Hypothetical protein*, *conserved*	*N*.*D*.	
Lin32H7	-2.41	-1.3 ± 0.0	0.011	0	0	a	LinJ.27.2500	Glycosomal phosphoenolpyruvate carboxykinase, putative.	+	-3.4 ± 0.2*
Lin33C5	1.75	0.8 ± 0.1	0.029	0	0	a	LinJ.35.3060	Glyoxylase I	N.D.	
Lin43B1	-3.04	-1.6 ± 0.2	0.005	6e-78	5e-178	b	LinJ.27.2480	60S acidic ribosomal protein	N.D.	
Lin54G3	1.73	0.8 ± 0.1	0.029	0	0	b	*LinJ24*.*1230*	*Hypothetical protein*, *conserved*	*N*.*D*.	
							LinJ.24.1240	Translation factor SUI1, putative	+	2.0 ± 0.4*
Lin68A9	1.91	0.9 ± 0.2	0.035	0	4e-85	b	LinJ.23.0140	Cyclophilin, putative	+	5.3 ± 0.3*
							*LinJ*.*23*.*0150*	*Vacuolar type proton translocating pyrophosphatase*	-	*1*.*4 ± 0*.1*
Lin95A10	-2.59	-1.4 ± 0.3	0.027	1e-152	2e-148	a	LinJ.27.2510	DEAD box helicase-like protein	N.D.	
Lin101B5	1.97	1.0 ± 0.6	0.049	0	0	b	LinJ.09.0960	Serine peptidase E, family S51	+	3.8 ± 0.1*
Lin101D5	-2.15	-1.1 ± 0.1	0.005	0	9e-37	b	LinJ.31.1680	GINS complex subunit Psf3	N.D.	
Lin123D6	1.77	0.8 ± 0.1	0.001	0	0	b	LinJ.34.2660	Amastin-like protein	N.D.	
Lin138C1	-1.76	-0.8 ± 0.1	0.028	0	0	b	*LinJ*.*24*.*1360*	*Hypothetical protein*, *conserved*	*N*.*D*.	
							*LinJ*.*24*.*1370*	*Hypothetical protein*, *conserved*	*N*.*D*.	
							*LinJ*.*24*.*1380*	*Translation initiation factor 2*	-	*-1*.*1 ± 0*.*4*
Lin150A5	-3.10	-1.6 ± 0.4	0.033	9e-177	0	b	*LinJ*.*30*.*2310*	*Hypothetical protein*, *conserved*	*N*.*D*.	
							*LinJ*.*30*.*2320*	*Hypothetical protein*, *conserved*	*N*.*D*.	
							*LinJ*.*30*.*2330*	*Hyptothetical protein*, *conserved*	*N*.*D*.	
Lin169D1	2.69	1.4 ± 0.1	0.031	0	0	a	LinJ.26.0030	Methylmalonyl-CoA epierase	+	10.0 ± 0.5*
Lin186B7	-1.9	-1.0 ± 0.1	0.003	0	0	a	LinJ.23.1220	Hydrophilic acylated surface protein B	+	2.7 ± 0.2*
Lin212F11	2.66	1.4 ± 0.2	0.006	0	0	b	LinJ.19.0940	4-coumarate-CoA ligase	+	7.3 ± 0.4*
Lin243E5	1.70	0.8 ± 0.1	0.003	0	0	a	LinJ.32.0400	Proteasome non-ATPase regulatory subunit 8	N.D.	
Lin247D7	2.36	1.2 ± 0.4	0.048	0	0	a	LinJ.28.0110	Proteasome beta subunit 3	N.D.	
Lin247G6	1.70	0.8 ± 0.1	0.00700	0	0	b	LinJ.29.2560	Heat shock protein 20	+	2.9 ± 0.2*
Lin250C3	1.74	0.8 ± 0.1	0.018	0	0	a	LinJ.32.2420	p-nitrophenylphosphatase	N.D.	
Lin252F9	2.01	1.0 ± 0.2	0.003	0	0	b	LinJ.30.2040	Calpain-like cysteine peptidase, Clan CA, family C2	N.D.	
Lin253D4	2.24	1.2 ± 0.5	0.001	0	0	b	LinJ.34.2660	Amastin-like protein	N.D.	
Lin268C9	2.24	1.2 ± 0.6	0.045	0	0	b	LinJ.01.0490	Fatty acyl-CoA synthetase 1	N.D.	
Lin297H12	3.67	1.9 ± 0.1	0.031	0	0	b	LinJ.35.4270	Hypothetical protein, conserved	N.D.	
Lin299F5	1.72	0.8 ± 0.1	0.027	0	2e-16	a	*LinJ*.*23*.*0570*	*Hypothetical protein*, *conserved*	*N*.*D*.	
							*LinJ*.*23*.*0580*	*Hypothetical protein*, *conserved*	*N*.*D*.	
Lin302C8	1.89	0.9 ± 0.0	0.001	0	0	b	LinJ.29.0930	Hypothetical protein, conserved	N.D.	
Lin305D10	1.89	0.9 ± 0.3	0.025	0	0	b	LinJ.23.0390	Hypothetical protein, conserved	N.D.	
Lin307A8	1.70	0.8 ± 0.1	0.022	-	0	c	*LinJ*.*09*.*0220*	*Hypothetical protein*, *conserved*	*N*.*D*.	
Lin309D1	1.89	0.9 ± 0.2	0.015	0	0	a	*LinJ*.*05*.*0060*	*Major vault protein*	-	*-1*.*0 ± 0*.*1*
							LinJ.05.0070	Dynein light chain, putative	N.D.	

### The differentially regulated metabolic genes up-regulated under serum depletion code for enzymes catalyzing rate-limiting reactions

The glycosomal phosphoenolpyruvate carboxykinase (gPEPCK) and the arginase are down-regulated under HIFBS-depletion, whereas the genes coding for the 4-coumarate-CoA ligase (4CCL), the fatty acyl-CoA synthetase 1 (FAS1), the methionine synthase reductase (MTRR), the p-nitrophenylphosphatase (PNPP) and the methylmalonyl-CoA epimerase (MMCE) are up-regulated.

### Branched-chain amino acid aminotransferase (BCAT) activity slightly but significantly decreases in HIFBS-depleted promastigotes

**Fig 6:**
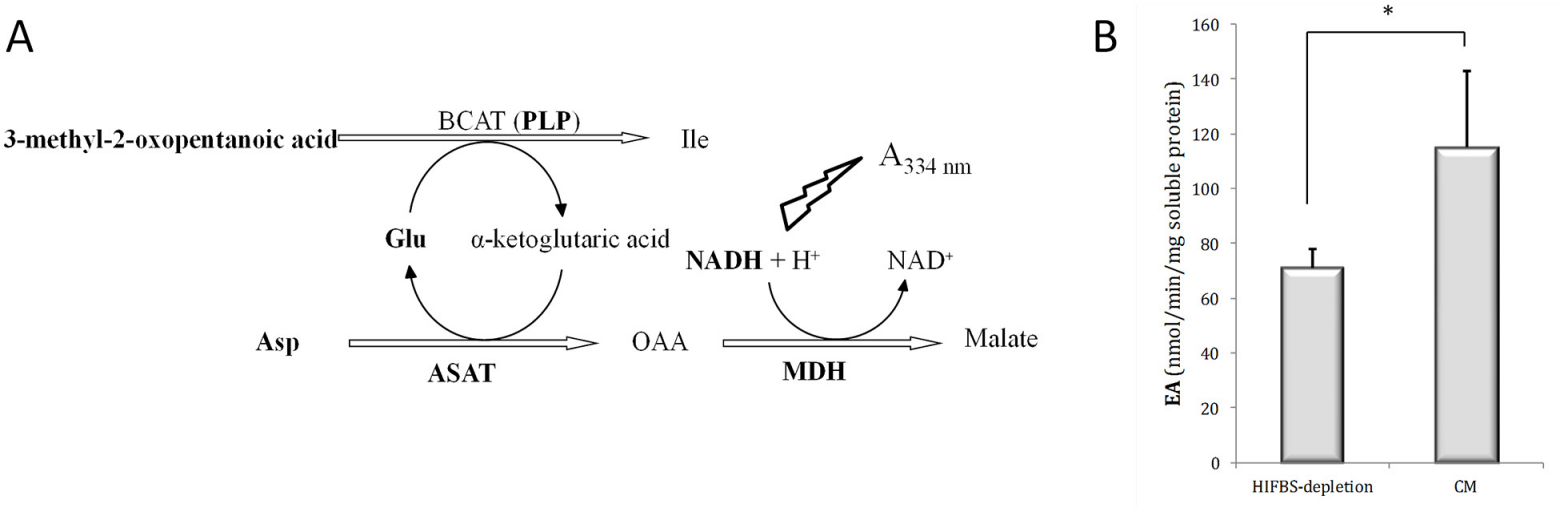
Enzyme activity of branched chain amino transferase in total protein extracts. (A) Basis of the BCAT assay. Enzymes and reagents added to the protein extracts are represented in bold. Ile transamination is reversible and the α-ketoglutarate is the amino acceptor. In this assay, the reverse reaction takes place because the 3-methyl-2-oxopentanoic acid is provided as the amino acceptor and Glu as the donor. This reaction is coupled to Asp transamination by adding this substrate and the ASAT. The product of this reaction (OAA) is reduced to malate by the MDH in the presence of the coenzyme NADH. The BCAT activity is indirectly measured as NADH oxidation (absorbance decay at 334 nm). Abbreviations: ASAT, L-aspartate aminotransferase; Glu, glutamic acid; MDH, malate dehydrogenase; PLP, pyridoxal phosphate. (B) BCAT activity in protein extracts of HIFBS-depleted and CM promastigotes. Three biological replicates were performed. HIFBS-depletion significantly decreases the specific BCAT enzymatic activity (EA) (t-test, p* = 0.022 < 0.05). Means and standard deviation (SD) of the EA are provided.

BCAT activity was measured using 3-methyl-oxopentanoate as the substrate and glutamate as the co-substrate (Fig 6A) in direct relation to NADH (A_334nm_) decay (S2 Table). Background activity was not observed in negative control reactions set in the absence of NADH. The enzyme activity (EA) measured as nmol/min/mg soluble protein is about 1.6-fold higher in HIFBS-depleted promastigotes that in reference CM promastigotes (Fig 6B). The differences observed are statistically significant (paired Student's t test, p = 0.022).

## Discussion

The presence of complement proteins contained in serum drastically decreases viability of promastigotes. In fact, only about 3% of stationary phase promastigotes survive when they are transferred to normal human serum [25]. Therefore, heat inactivation of serum is essential for appropriate culturing of *Leishmania* promastigotes.

Serum provides complex nutrients for appropriate growth, despite heat inactivation affects thermolabile nutrients such certain vitamins and amino acids. However, complete medium for promastigote culturing includes the defined medium RPMI 1640, which contains all protein amino acids and required vitamins. Some proteins are probably denatured at 56°C, whereas some others are not (e.g. albumin, which coagulates at higher temperatures). Lipids are denatured at higher temperatures than proteins. In fact, the major effect observed below the denaturation temperature range is not conformational transition of a single molecule but a change in the conformation of the supramolecular structure [26, 27]. For example, lipid monolayers of bilayers may be disintegrated without a conformational change in each single molecule, i.e. the gel-to-liquid transition due to temperature increase. Anyway, both native and denatured proteins and lipids are also source of their backbone residues as nutrients. Color does not change either, which indicates that the Fe-hemin complex is mantained intact after heating at 56°C. This is very important, as Fe is essential for proper growth of promastigotes.

HIFBS-depleted promastigotes are not differentiated to an amastigote-like stage, as revealed by morphology and constant abundance of the LACK protein (Fig 4 and S3 Table) and the A2 transcript (S3 Table). The reduced growth rate of HIFBS-depleted promastigotes (Fig 1A) may be related with the down-regulation of the GINS Psf3 gene (Fig 5, Table 1). The relationship between this expression profile and tetraploidy (Fig 1B) might be explained by hypothetical impairment of mitosis with cytokinesis. The G1 and G2 peaks are slightly displaced respectively from the 200 and 400 values in the FL2 axis in the case of CM and from the 400 and 800 values in the HIFBS-depletion plot (Fig 1B). This is consistent with constitutive aneuploidy observed in the genus *Leishmania* [28–30].

An unusually low differential expression rate has been reported in all the stages of different *Leishmania* species compared to other organisms [31–33]. In this context, the differential gene expression rate of HIFBS-depleted promastigotes is even more reduced than usual compared to CM control promastigotes. However, HIFBS-depletion affects important metabolic genes involved in limiting or crucial steps. One of them is the gPEPCK, which is down-regulated not only by HIFBS depletion, but also in metacyclic promastigotes isolated from the stomodeal valve of the sand fly (unpublished result), in amastigotes with respect to cultured promastigotes [31] and by the specific effect of temperature increase plus acidification towards differentiation [34]. These stages and experimental conditions involve nutrient depletion. The glycolytic genes remain constantly expressed at the transcript level in HIFBS-depleted promastigotes and gluconeogenesis is probably less active due to gPEPCK down-regulation. One of the most important energy and carbon sources for amastigotes is glucose obtained from the host, although glucolysis is more active in promastigotes in the absence of starvation and β-oxidation of fatty acids in amastigotes [35]. RPMI is a rich medium containing plenty of glucose (11 mM), which is in agreement with the gPEPCK expression profile found because apparently, gluconeogenesis is not required under these conditions. In contrast, the FAS1 gene is up-regulated in HIFBS-depleted promastigotes, probably because of the absence of the complex lipoid substances provided by serum. MMCE up-regulation suggests that the rate of branched chain amino acid and/or odd-chain fatty acid degradation is higher in HIFBS-depleted promastigotes than in CM. In fact, promastigotes and amastigotes are able to use amino acids as their major carbon sources [15]. No gene directly involved in branched-chain amino acid catabolism is differentially regulated in HIFBS-depleted promastigotes, whereas the MMCE gene is involved in both branched-chain amino acid and odd-chain fatty acid lipid catabolism [36]. For this reason, we aimed to evaluate the BCAT activity as an indication of relative activity of branched-chain amino acid degradation in culture (HIFBS-depletion versus CM promastigotes). The BCAT activity decreases under serum depletion in promastigotes (Fig 6B and S1 Fig). This finding suggests that MMCE up-regulation is not related to an increase of activity of branched chain amino acid catabolism but to degradation of odd-chain fatty acids. This activity may be linked to the 4CCL. These findings suggest that odd-chain fatty acids may be degraded to allow the biosynthesis of common fatty acids that may be required under depletion of complex lipids contained in serum. Methionine biosynthesis would be also required in HIFBS-depleted promastigotes, as the MTRR is up-regulated in these conditions. The cofactor S-adenosylcobalamine is oxidized over time when it is coupled to the methionine synthase, thus inactivating its activity. The role of the MTRR is keeping the methionine synthase (MTR) active by reverting oxidation of the cofactor. In addition to the amino acids provided in the HIFBS-depleted medium (i.e. RPMI), a possible source would be protein turn-over via the ubiquitin proteasome, which is suggested on the basis of the up-regulation of PSMD8 and PSMB3. To summarize, according to the gene expression profiles, catabolism of sugar, fatty acids and amino acids may remain constant under serum depletion, whereas fatty acid and methionine biosynthesis may be favored and gluconeogenesis may decrease.

The PNPP-encoding gene is up-regulated in HIFBS-depleted promastigotes, as well as in axenic amastigotes with respect to promastigotes [37, 38]. The PNPP participates in this cycle and bears the phosphoglycolate phosphatase activity (E.C.3.1.3.18). The glyoxylate cycle is present in *Leishmania* spp. and is probably related with glucolysis, gluconeogenesis and glycine biosynthesis [39]. In this case, PNPP up-regulation may be linked to glucolysis rather than gluconeogenesis because the gPEPCK is down-regulated in HIFBS-depleted promastigotes. Additionally, it may favor survival under nutrient depletion, as the glyoxylate pathway may be present in *Leishmania* to accelerate oxidative catabolism, given the low efficiency of the Krebs cycle in these organisms.

As a consequence of the unusual gene expression mechanisms in *Leishmania* spp. [40–45], post-transcriptional, translational and post-translational regulation are specially important processes in these organisms. The translation factor SUI1 is one of the up-regulated genes in HIFBS-depleted promastigotes. This gene was also found to be up-regulated in amastigote-like forms obtained by increasing temperature and lowering pH [34] but probably not by the presence of heavy metals (e.g. cadmium [46]) Therefore, this translation factor may influence translation control under specific stress situations like nutrient stress, temperature decrease and acidification. The hsp20 and the cyclophilin might participate in post-translational regulation processes provided their up-regulation in HIFBS-depleted promastigotes. The GLO1 is located in the kinetoplast and it is essential for survival. This gene is involved in the ketoaldehyde detoxification pathway. The up-regulation of the GLO1 gene under serum depletion may be one of the mechanisms the parasite displays to maintain certain growth rate, which is actually much slower than in the CM control (Fig 1A).

It has been described that nutrient depletion is associated to metacyclogenesis (reviewed in [5, 6]). Therefore, up-regulation of the HASPB and the amastin LinJ.34.2600 under serum depletion suggests that the differentiation state of HIFBS-depleted promastigotes is more advanced. In fact, the HASPB is associated to differentiation of promastigotes [47] and amastins constitute a superfamily of proteins of unknown function basically expressed in the amastigote stage (reviewed by [48]). However, the following considerations are in disagreement with a more advanced differentiation stage of promastigotes under serum depletion: i) morphology (i.e. higher frequency of stumpy instead of slender promastigotes); ii) the differentiation process encompasses proper growth in culture as well as within the sand fly gut it mimics, which is not observed in HIFBS-depleted promastigotes (Fig 1A); iii) ploidy alteration (Fig 1B); iv) down-regulation of the arginase in HIFBS-depleted promastigotes (Table 1); and v) decreased infectivity *in vitro* (Fig 2). High expression levels of the arginase increases the chance of survival of amastigotes within the host phagocytes [49]. The pre-adaptation hypothesis is essential to understand amastin and arginase expression in promastigotes. This hypothesis consists of a phenotype prepared in advance for differentiation of promastigotes to amastigotes, i.e. invasion of the mammalian host phagocyte [5, 6, 31, 50]. Therefore, an unsuccessful differentiation process takes place in HIFBS-depleted promastigotes, which are less infective than CM promastigotes. A possible explanation is the down-regulation of the arginase gene.

## Conclusions

In general, the axenic culture model is used to perform biological and biomedical studies concerning *Leishmania* promastigotes. As an insight into the role of inactivated serum in the culture medium, this study has revealed that serum depletion considerably decreases the growth rate of promastigote cultures and leads to reduced infectivity and ploidy alteration. Consequently, only mediums containing the complex nutrients of serum are appropriate in axenic cultures in order to mimic to some extent the natural developmental processes of promastigotes. The effect on the transcriptome is slight in terms of differentially regulation rate but important when gene function is considered (i.e. GINS Psf3, gPEPCK, FAS1, PNPP, MTTR, MMCE, HASPB, arginase and amastin). Down-regulation of the arginase in HIFBS-depleted promastigotes contributes to explain their reduced infectivity. The results discussed herein have provided clues to understand processes and to establish new hypotheses and observations that may be studied in the future. For example, the role of the glyoxylate cycle in these organisms or the elucidation of signal transduction pathways and their connection with stimuli and effector gene expression regulation mechanisms that are completely unknown in these organisms so far.

## Supporting Information

S1 Fig Decay of NADH concentration in the BCAT activity assay. One out of three biological replicates of the experiment is shown. (A) CM. (B) HIFBS-depletion.
(PPTX)

S1 Table qRT-PCR primers and probes. (XLS)

S2 Table Flow cytometry data. Data from one out of three biological replicates displayed in Fig 1B are shown for CM and HIFBS-depletion.
(PPT)

S3 Table Microarray control spots. HIFBS-depletion/CM hybridization results for positive and negative controls included in the genome microarrays.
(DOC)
