# Supplementary material for: Serum Removal from Culture Induces Growth Arrest, Ploidy Alteration, Decrease in Infectivity and Differential Expression of Crucial Genes in Leishmania infantum Promastigotes
Source: PLoS One. 2016 Mar 9;11(3):e0150172. doi: 10.1371/journal.pone.0150172 (PMC4784933; doi:10.1371/journal.pone.0150172)
Supplement: S1 Fig — One out of three biological replicates of the experiment is shown. (A) CM. (B) HIFBS-depletion. (PPTX) [file pone.0150172.s001.pptx]

## Slide 1
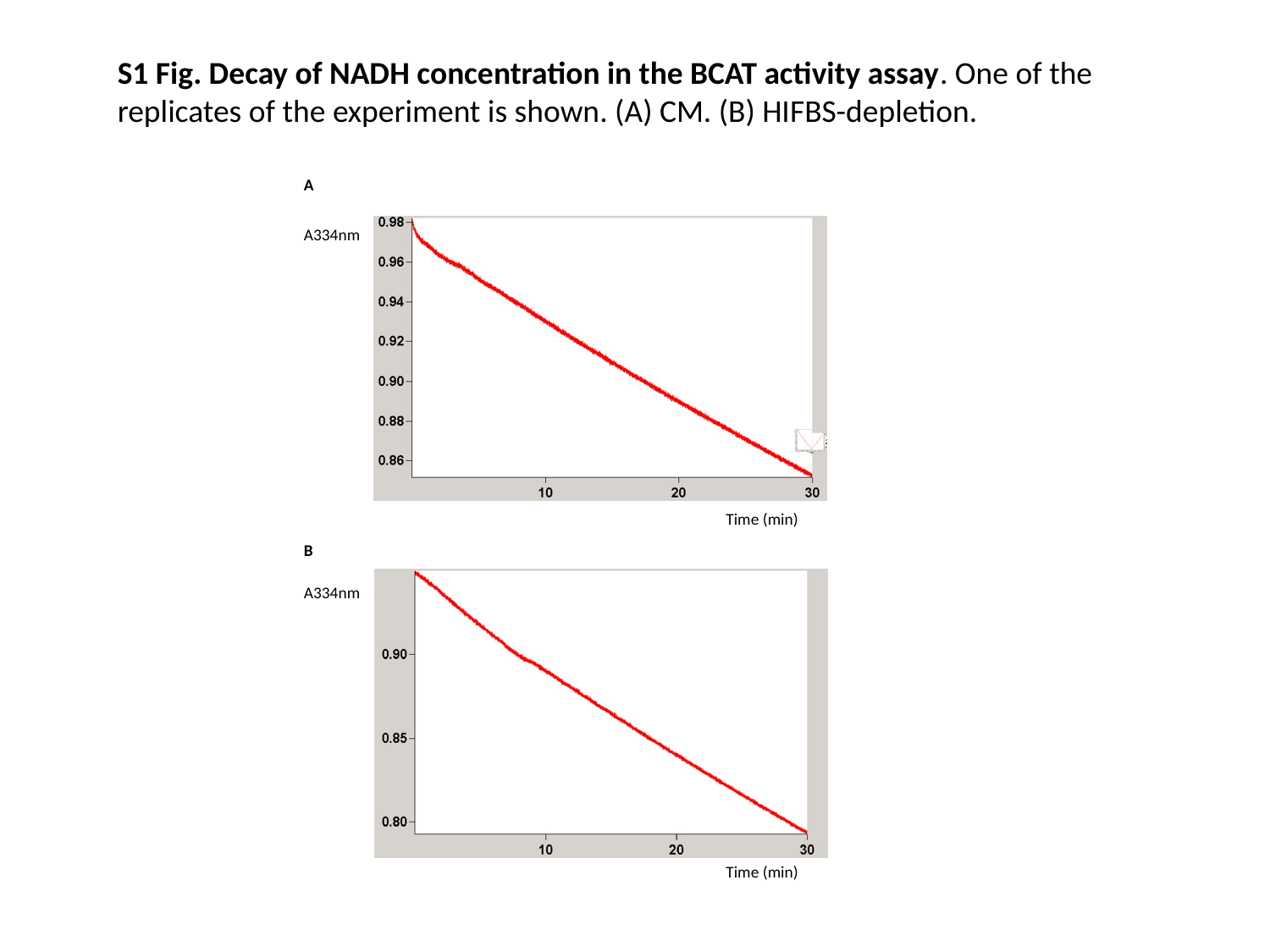

S1 Fig. Decay of NADH concentration in the BCAT activity assay. One of the replicates of the experiment is shown. (A) CM. (B) HIFBS-depletion.
