# Supplementary figures and images for: Serum Removal from Culture Induces Growth Arrest, Ploidy Alteration, Decrease in Infectivity and Differential Expression of Crucial Genes in Leishmania infantum Promastigotes
Source: PLoS One. 2016 Mar 9;11(3):e0150172. doi: 10.1371/journal.pone.0150172 (PMC4784933; doi:10.1371/journal.pone.0150172)

## Slide 1
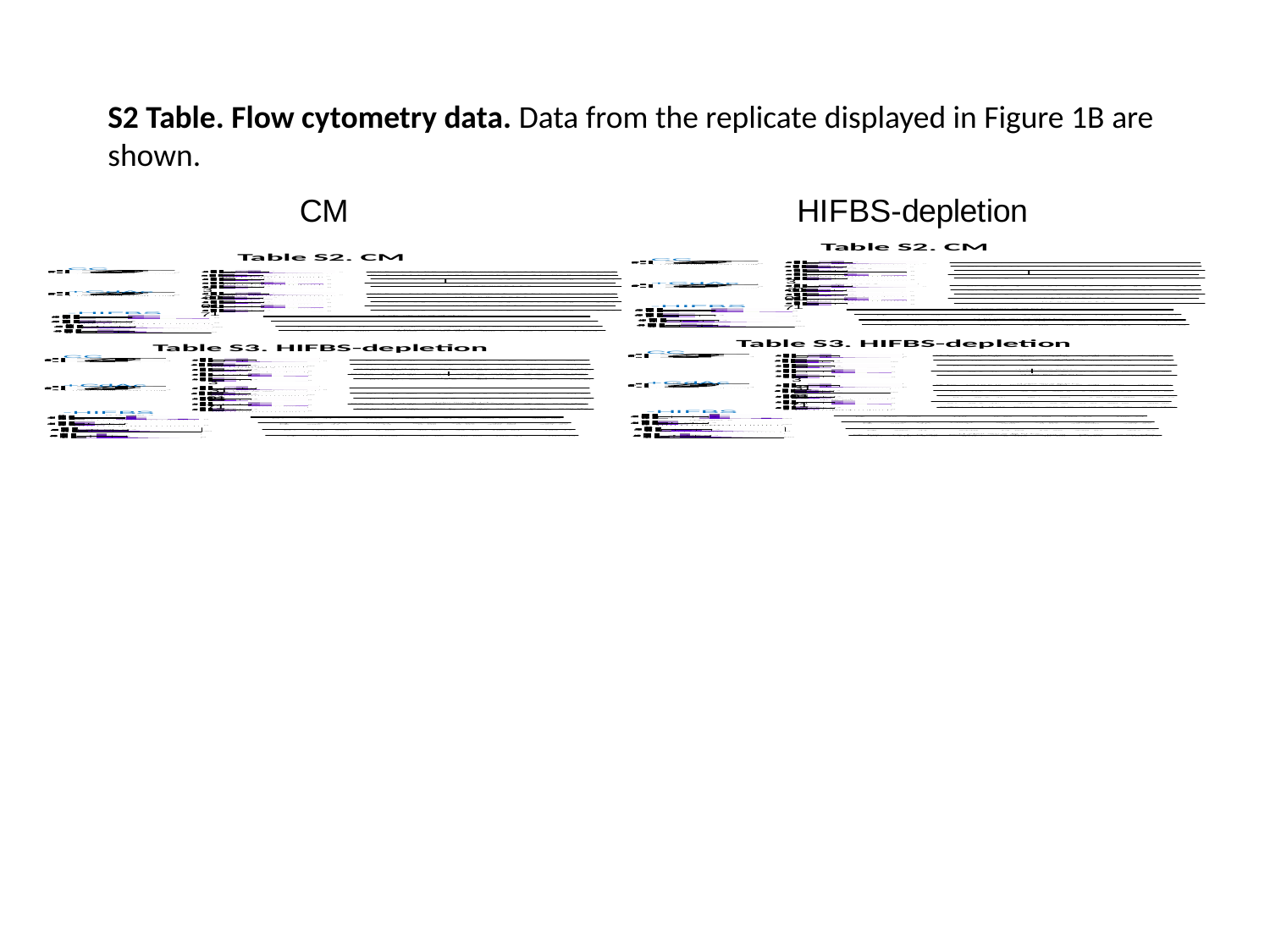

S2 Table. Flow cytometry data. Data from the replicate displayed in Figure 1B are shown.

Supplement: S2 Table — Data from one out of three biological replicates displayed in Fig 1B are shown for CM and HIFBS-depletion. (PPT) [file pone.0150172.s003.ppt]
