# Supplementary material for: Serum Removal from Culture Induces Growth Arrest, Ploidy Alteration, Decrease in Infectivity and Differential Expression of Crucial Genes in Leishmania infantum Promastigotes
Source: PLoS One. 2016 Mar 9;11(3):e0150172. doi: 10.1371/journal.pone.0150172 (PMC4784933; doi:10.1371/journal.pone.0150172)
Supplement: S3 Table — HIFBS-depletion/CM hybridization results for positive and negative controls included in the genome microarrays. (DOC) [file pone.0150172.s004.doc]

##### S3 Table. Results of the HIFBS-depletion/CM cDNA-DNA microarray hybridization analysis for positive and negative control spots. HIFBS-depletion/CM fold changes (F) and standard deviations (SD) are detailed, as well as p-value (α = 0.05). Absence of differential gene expression has been observed in positive controls (*p* ≥ 0.05). Mean fluorescence intensity (FU) and SD values are provided.

| ***Spot*** | | **F ± SD** | | ***p*** | | | | **Positive control** | |
| --- | --- | --- | --- | --- | --- | --- | --- | --- | --- |
| cLin79A1 | | 1.3 ± 0.0 | 0.332 | | | | *Li Polβ* | | |
| cLin79A2 | | -1.1 ± 0.1 | 0.054 | | | | *Li TopoII* | | |
| cLin79A3 | | 2.0 ± 0.1 | 0.007 | | | | *Li p36/LACK* | | |
| cLin79B1 | | 1.2 ± 0.3 | 0.268 | | | | *Li hsp70* | | |
| cLin79B2 | | 1.3 ± 0.3 | 0.565 | | | | *Ldo hsp70* | | |
| cLin79B3 | | 1.1 ± 0.1 | 0.860 | | | | *Lam hsp70* | | |
| cLin79C1 | | 1.1 ± 0.4 | 0.180 | | | | *Lma hsp70* | | |
| cLin79C2 | | 1.2 ± 0.1 | 0.166 | | | | *Li A2* | | |
| cLin79C3 | | -1.3 ± 0.5 | 0.830 | | | | *Ldo A2* | | |
| cLin79D1 | | 1.0 ± 0.0 | 0.145 | | | | *Li GAPDH* | | |
| cLin79D2 | | 1.4 ± 0.5 | 0.956 | | | | *Ldo GAPDH* | | |
| cLin79D3 | | 1.1 ± 0.2 | 0.913 | | | | *LigDNA* | | |
| cLin79H2 | | 1.1 ± 0.7 | 0.969 | | | | *Herring sperm DNA* | | |
| ***Spot*** | **Mean FU ± SD** | | | | **Negative control** | | | |  |
| cLin79E1 | | 51 ± 35 | | | | *Lfe nifA/hlyD* | | | |
| cLin79E2 | | 12 ± 3 | | | | *Lfe nifD/nifK* | | | |
| cLin79E3 | | 164 ± 74 | | | | *Lfe nifH* | | | |
| cLin79F1 | | 138 ± 35 | | | | *Lfe nifS/nifU* | | | |
| cLin79F2 | | 362 ± 127 | | | | *Lfe nifX/nifB* | | | |
| cLin79F3 | | 78 ± 26 | | | | *Lfe nifH/nifD* | | | |
| cLin79G1 | | 118 ± 57 | | | | *Lfe nifE* | | | |
| cLin79G2 | | 22 ± 12 | | | | *Lfe nifV/HesB* | | | |
| cLin79G3 | | 7 ± 1 | | | | *Lfe nifV* | | | |
| cLin79H1 | | 45 ± 21 | | | | *Lfe nifW/Bgene* | | | |
| cLin79H3 | | 27 ± 15 | | | | *1XSSC* | | | |
|  | |  | | | | | |  | |
